# Effect of topical nitroglycerin on neoangiogenesis and pedicle-independent viability in a rat dorsal skin flap model

**DOI:** 10.17305/bb.2025.12781

**Published:** 2025-08-25

**Authors:** Oğuzhan Karakoç, Selman Hakkı Altuntaş, İlkay Armağan

**Affiliations:** 1 Department of Plastic, Reconstructive and Aesthetic Surgery, Suleyman Demirel University, School of Medicine, Isparta, Türkiye; 2 Department of Histology, Faculty of Medicine, Suleyman Demirel University, School of Medicine, Isparta, Türkiye

**Keywords:** Flap autonomization, topical nitroglycerin, interpolation flaps, angiogenesis, rat flap model

## Abstract

Interpolated flaps are frequently used in reconstructive surgery when free tissue transfer is not feasible, but they require staged procedures due to pedicle dependence. Flap autonomization, the process by which transferred tissue develops new vascular connections and survives independently of its pedicle, is essential before division. Although methods such as delay techniques, hyperbaric oxygen (HBO), vascular endothelial growth factor (VEGF), and stem cell therapies have been tested to enhance angiogenesis, the effect of topical nitroglycerin (NTG), a nitric oxide (NO) donor with vasodilatory, anti-inflammatory, and angiogenic properties, has not been investigated. This study aimed to evaluate the effect of topical NTG on neoangiogenesis and flap autonomization in a rat dorsal skin flap model. Sixty Wistar-Albino rats were divided into five groups (*n* ═ 12). A 3×3 cm dorsal flap with a caudal pedicle was elevated in all animals. In Groups 1–3, pedicles were transected on day 5: Group 1 received vaseline, Group 2 received NTG for 5 days then vaseline, and Group 3 received NTG continuously. Groups 4 and 5 were sacrificed on day 5 to assess early angiogenesis after vaseline or NTG. Flap survival was analyzed with ImageJ, angiogenesis with VEGF, CD34, and CD105 staining, and histology with Hematoxylin and Eosin (H&E) and Masson’s Trichrome. Flap survival was significantly greater in Groups 2 (485.5 mm^2^) and 3 (757.3 mm^2^) than in Group 1 (273.5 mm^2^), with Group 3 highest (*P* < 0.01). NTG-treated groups showed increased VEGF, CD34, and CD105 expression, with the strongest angiogenesis in Group 3. Group 5 also had higher vascular proliferation than Group 4 (*P* < 0.001). Histology showed that NTG reduced epithelial disruption, hemorrhage, collagen degradation, and leukocytic infiltration while enhancing vascular proliferation. In conclusion, continuous topical NTG enhanced angiogenesis and accelerated flap autonomization, leading to greater viability after pedicle division. NTG may help shorten pedicle division intervals and improve outcomes in reconstructive surgery, but further molecular and clinical studies are needed.

## Introduction

Reconstructive surgery often serves as the primary intervention; however, certain patient conditions may necessitate the use of interpolated flaps for reconstruction. Interpolated flaps, a specific type of local flap, involve donor sites that are not contiguous with recipient sites, with healthy tissue situated between them. As a result, the pedicle must either remain beneath or, more commonly, on top of the intervening tissue. Most interpolated flaps are performed in at least two stages, which include necessary tissue revisions. Common examples of these flaps include the forehead flap, Abbe flap, Cutler-Beard flap, cross-finger flap, and pedicled groin flap.

The primary disadvantage of interpolated flaps is the requirement for multiple surgical stages. Nonetheless, they are preferred in cases where repair with similar tissue is desirable, when sufficient adjacent skin is unavailable at the defect, or when free flap repair is not feasible. For the closure of an open wound, a pedicle is not essential; it merely provides blood circulation to the tissue intended to cover the defect, and its presence can often be problematic for the patient. As the flap tissue undergoes angiogenesis in the recipient area, it becomes capable of surviving independently of the pedicle. At this point, the pedicle is no longer necessary and can be excised to achieve the desired outcome.

Flap autonomy, defined as pedicle-independent viability, refers to the process by which transferred tissue becomes independent of its original vascular supply. In a study examining this phenomenon, researchers observed that following ischemic preconditioning and transection of the pedicle of a superficial inferior epigastric artery-based skin flap on day 5, only 10% of the distal skin of the flap remained viable [1].

It is known that free flaps can survive despite issues with their vascular pedicles; however, very little is known about the precise impact of various factors—such as anatomical localization and internal or external conditions—or about the duration of the flap autonomization process [2, 3]. Furthermore, there is insufficient evidence regarding the occurrence of neoangiogenesis in all flaps and the extent to which these flaps remain reliant on their primary vascular pedicles over time [4]. The literature identifies several key factors influencing neovascularization, which can be broadly categorized into wound bed characteristics, flap location, and flap type. A variety of strategies have been explored to enhance neovascularization. These strategies include physical methods, such as the delay technique and intermittent pedicle compression, as well as experimental approaches, including L-theanine administration, hyperbaric oxygen (HBO) therapy, epinephrine injection into the pedicle, application of adipose-derived stem cells, treatment with epidermal growth factor (EGF), and administration of exogenous vascular endothelial growth factor (VEGF) [5–8].

Infections, tumors, radiation, and vascular insufficiency—factors commonly associated with atherosclerosis, smoking, and diabetes—can significantly impede the process of neoangiogenesis [9, 10].

Research on the therapeutic applications of nitric oxide (NO) continues to expand, driven by its capacity to activate various intracellular mechanisms. Numerous clinical studies have investigated the efficacy of nitroglycerin (NTG) in treating conditions such as anal fissures, tendinopathies, and preventing mastectomy skin flap necrosis. A rapid autonomization process, in which the flap establishes a vascular network independent of its pedicle, can mitigate the need for revision surgeries due to inadequate circulation at the pedicle or complications from free tissue transfer anastomoses. This process results in reduced flap loss, fewer complications associated with prolonged hospital stays, expedited patient recovery, and decreased healthcare costs. The primary objective of this study is to examine the effects of topical NTG on the duration of skin flap survival without pedicle dependency (flap autonomization) and the process of neoangiogenesis.

## Materials and methods

### Study design

In this study, *a priori* power analysis was conducted using G*Power 3.1 to estimate the minimum sample size necessary for a one-way ANOVA. Assuming a large effect size (*f* ═ 0.4), an alpha level of 0.05, and a power of 0.80, the total sample size required for five groups was determined to be 60 animals (*n* ═ 12 per group). Sixty female Wistar-Albino rats, each weighing between 250 and 300 grams, were sourced from the Süleyman Demirel University Animal Production and Experimental Research Laboratory. Ethical approval for this experimental study was granted by the Süleyman Demirel University Local Ethics Committee for Animal Experiments (Approval Date: 08/06/2023; Approval No: 06-178). Throughout the experiment, the rats were individually housed in separate cages within the same room, maintaining controlled environmental conditions (24 ^∘^C, 12-h light/dark cycle). They were provided with standard chow and water ad libitum.

To assess the microscopic and macroscopic effects of topical NTG on the flap, sixty rats were randomly assigned to five equal groups (*n* ═ 12 per group) utilizing a simple manual randomization process to eliminate selection bias.

**Figure 1.**
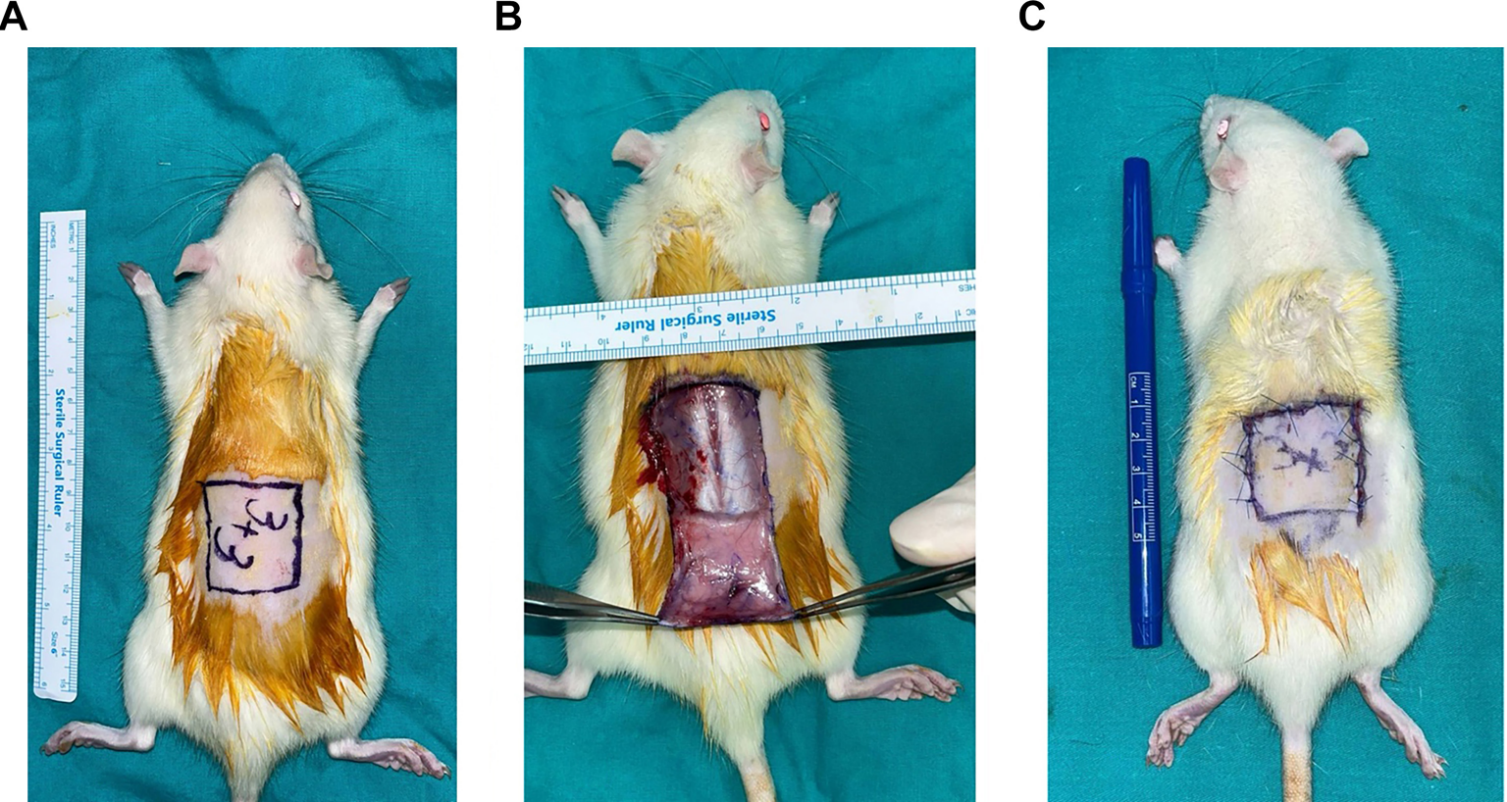
**Creation of the dorsal caudally pedicled skin-flap model in Wistar-Albino rats.** (A) Preoperative marking of a 3 × 3 cm dorsal skin flap; the pedicle is positioned between the caudal and iliac crests; (B) Elevation of the flap on its caudal pedicle; (C) Flap repositioning into the original bed and primary closure with interrupted 5-0 polypropylene sutures. The same standardized flap design and procedure were applied in all groups. Rulers indicate centimeters.

Prior to surgery, anesthesia was induced through intraperitoneal injection of 10 mg/kg xylazine and 30 mg/kg ketamine. In all groups, 3×3 cm skin flaps were designed with a caudal pedicle, ensuring that the pedicle remained between the caudal and iliac crests. The flap tissue was elevated, repositioned, and secured with 5.0 polypropylene sutures (Figure 1). Immediately following surgery, the animals were placed under an infrared heating lamp for 30 min to maintain postoperative warmth. They were then returned to their cages and provided ad libitum access to food and water. Behavioral monitoring was conducted daily, revealing no signs of distress, reduced activity, or feeding abnormalities. All histological evaluations and ImageJ-based analyses were performed by investigators blinded to the treatment groups.

### Experimental groups

The first three groups were assessed both clinically and microscopically. On the fifth day post-flap elevation, the pedicles of the flaps were transected to facilitate the development of a new capillary network for improved circulation. One week after pedicle transection, biopsies were collected for microscopic evaluation, and photographs were taken for macroscopic assessment.

**Group 1**: Vaseline was applied for five days (until pedicle transection) and continued for an additional seven days thereafter.

**Group 2**: Topical NTG was administered for five days (until pedicle transection), followed by Vaseline for the subsequent seven days.

**Group 3**: Topical NTG was applied continuously for five days (until pedicle transection) and for seven days following transection.

In addition to the clinical evaluation groups, two additional groups were included to assess tissue vascularization on the fifth day—prior to pedicle transection. The vascular network of the flaps on day five was evaluated in these groups:

**Group 4**: Vaseline was applied for five days following flap elevation.

**Group 5**: Topical NTG was applied for five days following flap elevation.

In Groups 4 and 5, the animals were sacrificed on day five, and tissue vascularization was evaluated microscopically.

### Topical NTG application

The topical NTG utilized in this study was a 5% ointment sourced from the Turkish Magistral Pharmacists Association. The application was conducted using the Finger Tip Unit (FTU) method, where one FTU is approximately equivalent to 0.5 g of ointment. For each application, one-third of an FTU (approximately 0.17 g) of the 5% NTG ointment was administered every 12 h, delivering a dosage of approximately 8.5 mg of NTG per application. The ointment was uniformly distributed over the entire flap surface, which measured 3 cm × 3 cm (9 cm^2^), resulting in an estimated dose of approximately 0.94 mg/cm^2^ per application.

### Clinical evaluation

**Figure 2.**
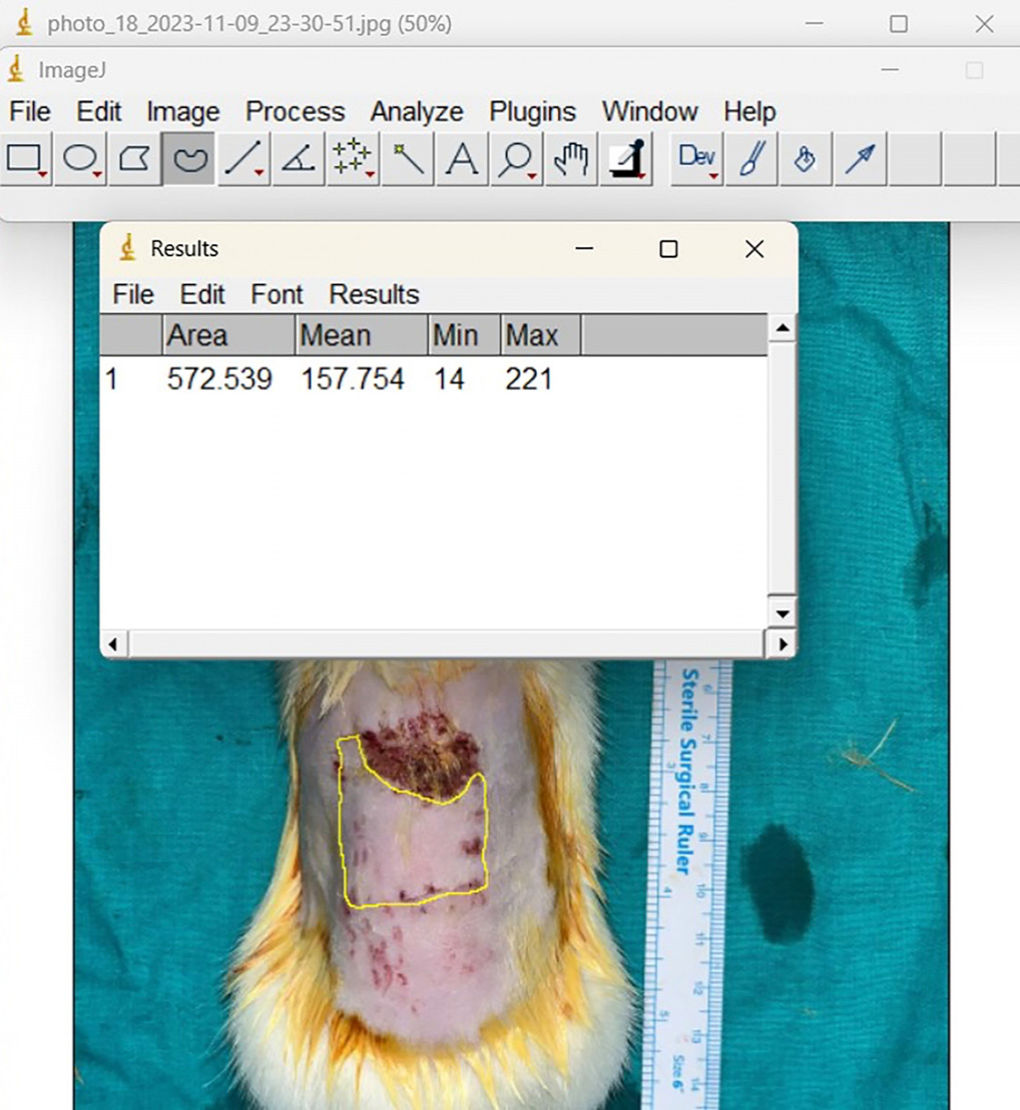
**Quantification of viable flap area (mm^2^) using ImageJ.** Representative planimetric analysis performed on day 7 after pedicle transection, when necrosis was considered final. A metric ruler within the photograph was used to calibrate scale. The viable (non-necrotic) portion of the flap was traced with a polygon ROI (yellow), and ImageJ (open-source Java software, USA) returned the area in square millimeters (Results window). For each animal, percentage viability was calculated as (viable area ÷ total flap area) × 100.

On the seventh day following pedicle transection, the necrotic areas were considered final. For macroscopic evaluation, the ratio of the viable flap area (excluding necrotic regions) to the total flap area was calculated. Measurements were conducted in square millimeters using the ImageJ program, an open-source Java image processing software (USA), with a ruler incorporated in the photographs taken of the rats (Figure 2).

### Histological analysis

For microscopic evaluation, a single biopsy specimen comprising the middle portion of the flap edge and adjacent normal skin was collected from each rat. The excised tissues were placed under running water overnight to remove the fixative, followed by dehydration in six different alcohol solutions of increasing concentration (ranging from 50% to absolute) for one hour each. The tissues were then cleared in xylene for 10–15 min, infiltrated with paraffin in an oven at 60 ^∘^C for three hours, and embedded in paraffin blocks. From these blocks, 4 µm thick sections, encompassing all skin layers parallel to the long axis of the tissue sample, were obtained using a rotary microtome (Leica RM2155 RT, Nussloch, Germany).

For immunohistochemical analysis of endothelial proliferation, three primary antibodies—VEGF, CD34, and CD105 (dilutions: 1:100, 1:100, and 1:50, respectively; Elabscience, TX, USA)—were employed. Sections measuring 4 µm in thickness, derived from deparaffinized tissue blocks, were affixed to polylysine-coated slides. Deparaffinization was achieved through overnight drying at 60 ^∘^C and two 20-min treatments with xylene. Subsequent rehydration occurred via a gradient of decreasing alcohol concentrations using phosphate-buffered saline (PBS). Antigen retrieval involved microwaving the sections in a 10% citrate buffer for 2 min, followed by a 20-min cooling period. The peripheries of the sections were delineated with a Pappen pen, and endogenous peroxidase activity was inhibited using 3% hydrogen peroxide (H_2_O_2_). Following protein blocking, the sections were incubated with the VEGF, CD34, and CD105 primary antibodies for 1 h at room temperature, then overnight at +4 ^∘^C within a humid chamber. After secondary antibody incubation, the sections were treated with streptavidin-HRP, developed using diaminobenzidine (DAB) chromogen, counterstained with Mayer’s Hematoxylin, mounted with Entellan, and assessed with a digital microscope. In two randomly selected sections per animal, five random fields at ×400 magnification were analyzed, and all vessels exhibiting immunoreactivity were counted, regardless of size; averages were subsequently calculated.

Additionally, five parameters were assessed through Hematoxylin and Eosin (H&E) and Masson’s Trichrome staining: disruption of epithelialization, hemorrhage, collagen degradation, vascular proliferation, and leukocytic infiltration. Each parameter was scored on a semi-quantitative scale: 0 = absent, 1 = mild, 2 = moderate, 3 = severe, and 4 = very severe. For each rat, two randomly selected tissue sections were examined, and five randomly chosen fields at ×200 magnification were evaluated per section. The mean score of these five fields was calculated for each parameter.

### Statistical analysis

Data were analyzed using SPSS Version 26.0 (IBM Corp., Armonk, NY, USA). Normality was evaluated with the Shapiro–Wilk test. For normally distributed variables, descriptive statistics are reported as mean ± standard deviation and analyzed using one-way ANOVA, followed by Bonferroni post-hoc corrections. For non-normally distributed data, the Kruskal–Wallis test was utilized, accompanied by Dunn’s post-hoc test with Bonferroni adjustment. A type I error rate of 5% was established for all analyses, with adjusted *P* values <0.05 deemed statistically significant.

## Results

### Clinical findings

**Figure 3.**
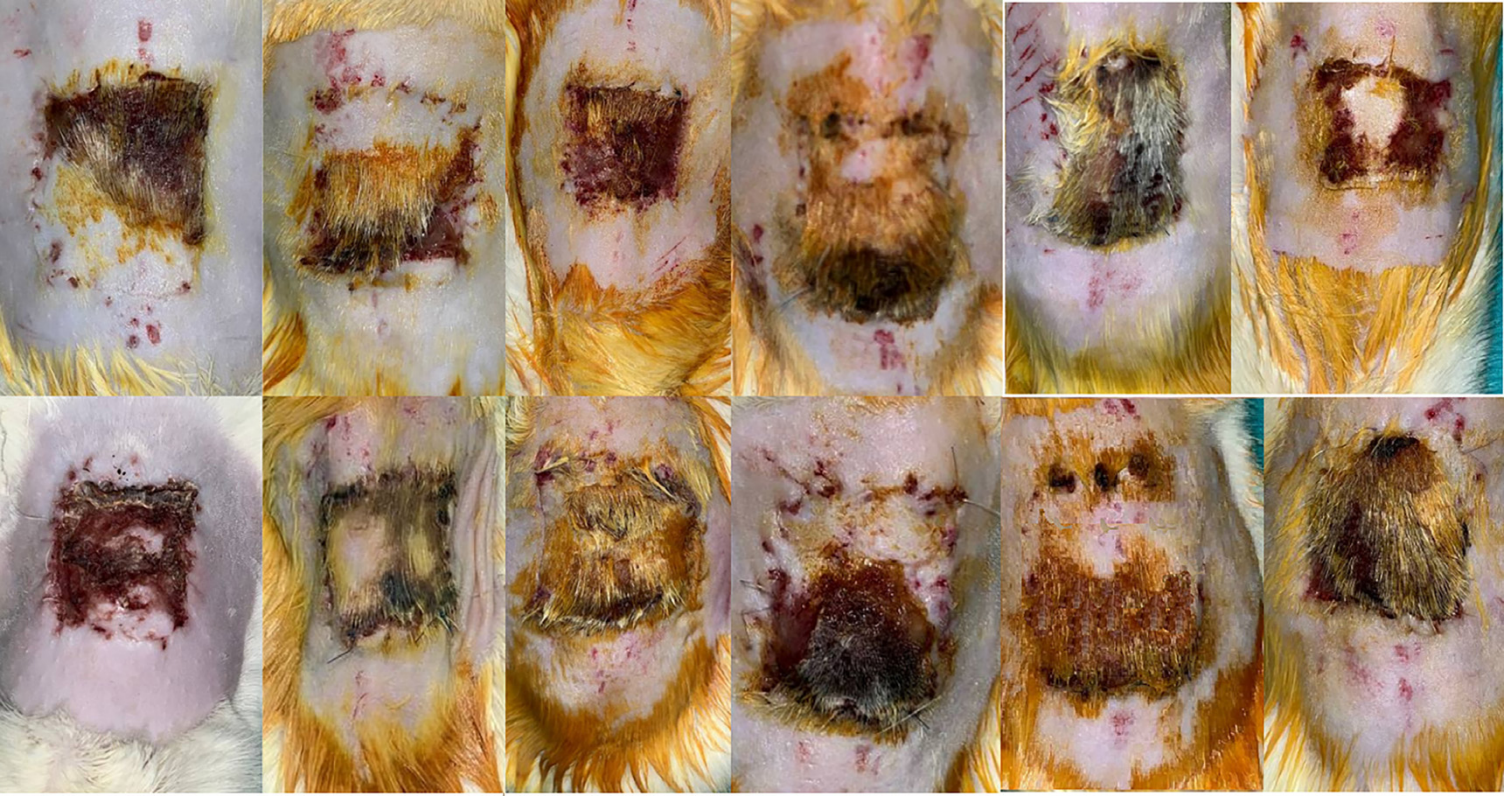
**Surviving areas of flaps in Group 1 on the 7th postoperative day after pedicle division.** The viable regions are markedly smaller compared to the other experimental groups, showing extensive necrosis and limited tissue survival.

**Figure 4.**
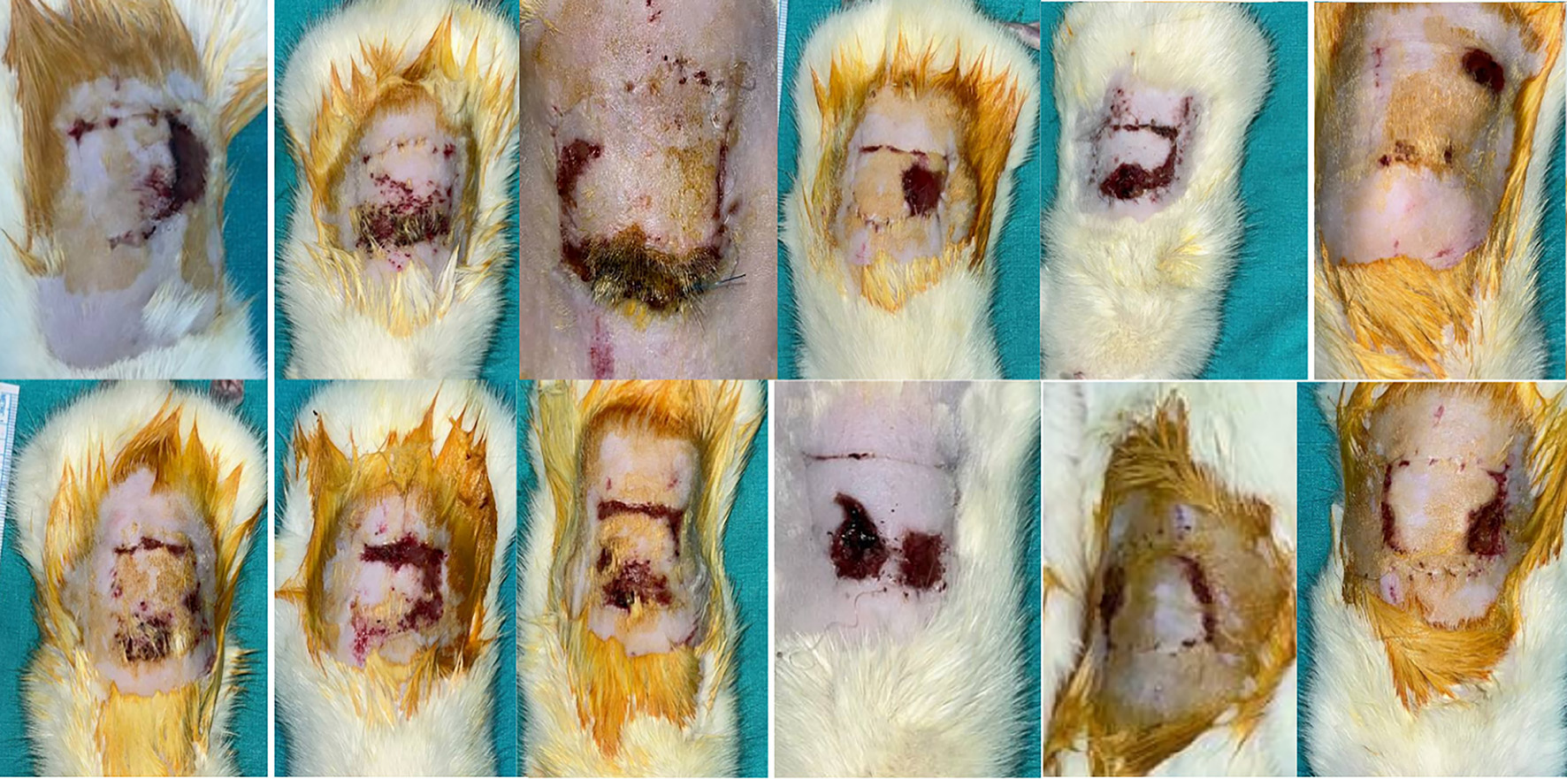
**Representative appearance of surviving flap areas in Group 2 on the 7th postoperative day after pedicle division**. The extent of viable tissue is greater than in Group 1, but remains smaller when compared with Group 3.

**Figure 5.**
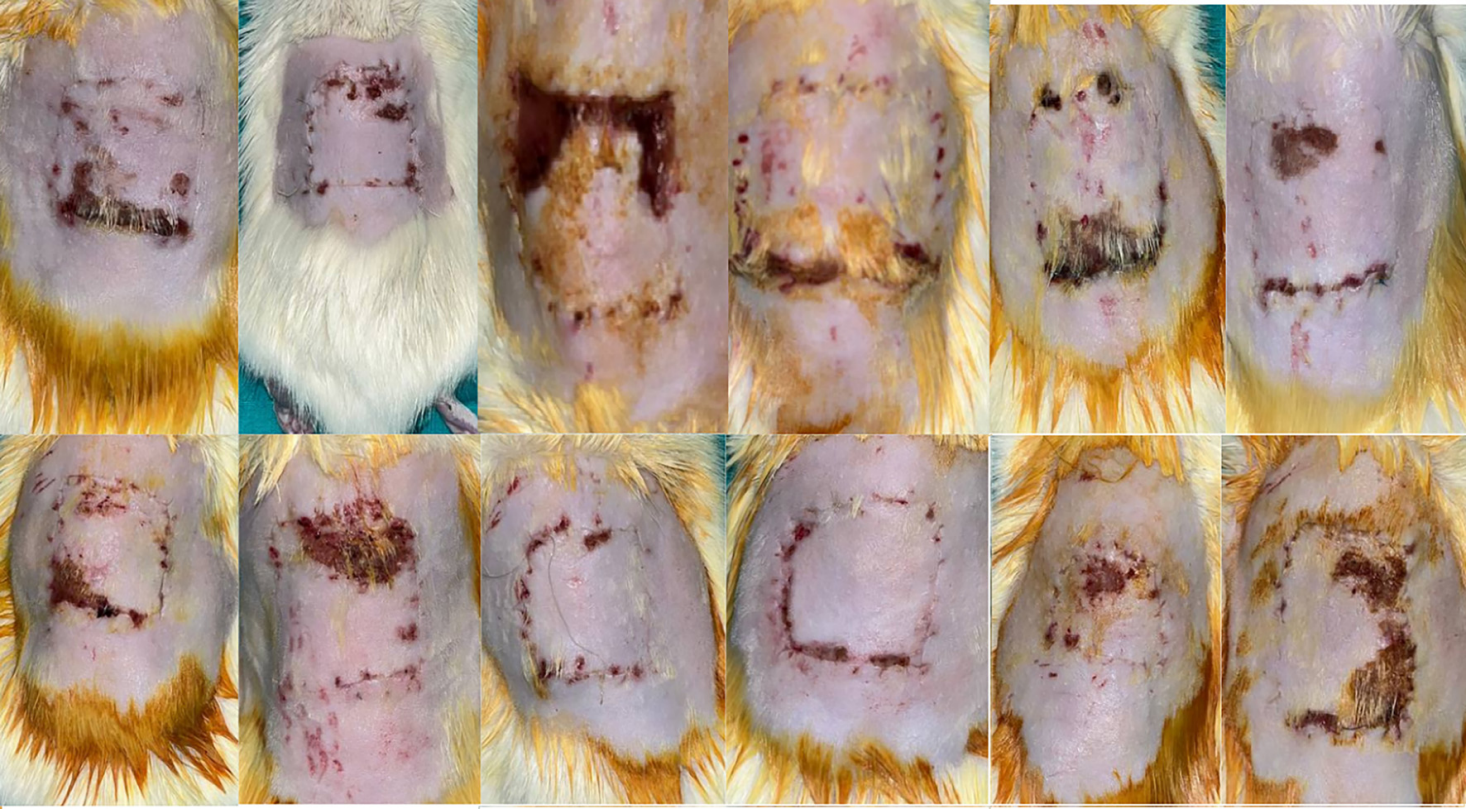
**Representative appearance of surviving flap areas in Group 3 on the 7th postoperative day after pedicle division.** A visibly larger viable region is present compared with both Group 1 and Group 2.

**Figure 6.**
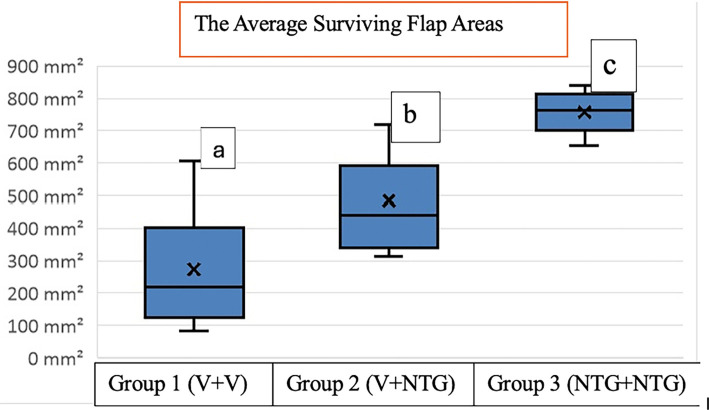
**Box-plot representation of average surviving flap areas in the three experimental groups on the 7th postoperative day**. Groups marked with different letters differ significantly. Abbreviations: V: Vehicle; NTG: Nitroglycerin.

The average surviving flap areas were as follows: Group 1 (5 days of TV + 7 days of TV) had an average surviving area of 273.5, Group 2 (5 days of TN + 7 days of TV) had 485.5, and Group 3 (5 days of TN + 7 days of TN) had 757.25. Statistical analysis indicated that Group 3 exhibited a significantly larger surviving area compared to both Group 1 (*P* < 0.001) and Group 2 (*P* < 0.001). Additionally, a significant difference was observed between Groups 1 and 2 (*P* ═ 0.002) (see Table 1 and Figures 3–6).

**Table 1:** Mean surviving flap areas (mm^2^) and their percentages, presented with standard deviations and 95% confidence intervals

	**Average surviving areas (mm^2^)**	**95% CI**		**Adjusted *P* values**
Group 1	273,5 (%30,40) ±176,74	161,2–385,8	Group 1-2	0.002
Group 2	485,5 (%53,94) ±147,67	391,6–579,3	Group 1-3	<0.001
Group 3	757,25 (%84,14) ±61,40	720,5–830,6	Group 2-3	<0.001

Adjusted *P* values represent pairwise comparisons between groups following one-way ANOVA with Bonferroni correction. A *P* value < 0.05 was considered statistically significant. Abbreviation: CI: Confidence interval.

### Immunohistochemical findings

**Figure 7.**
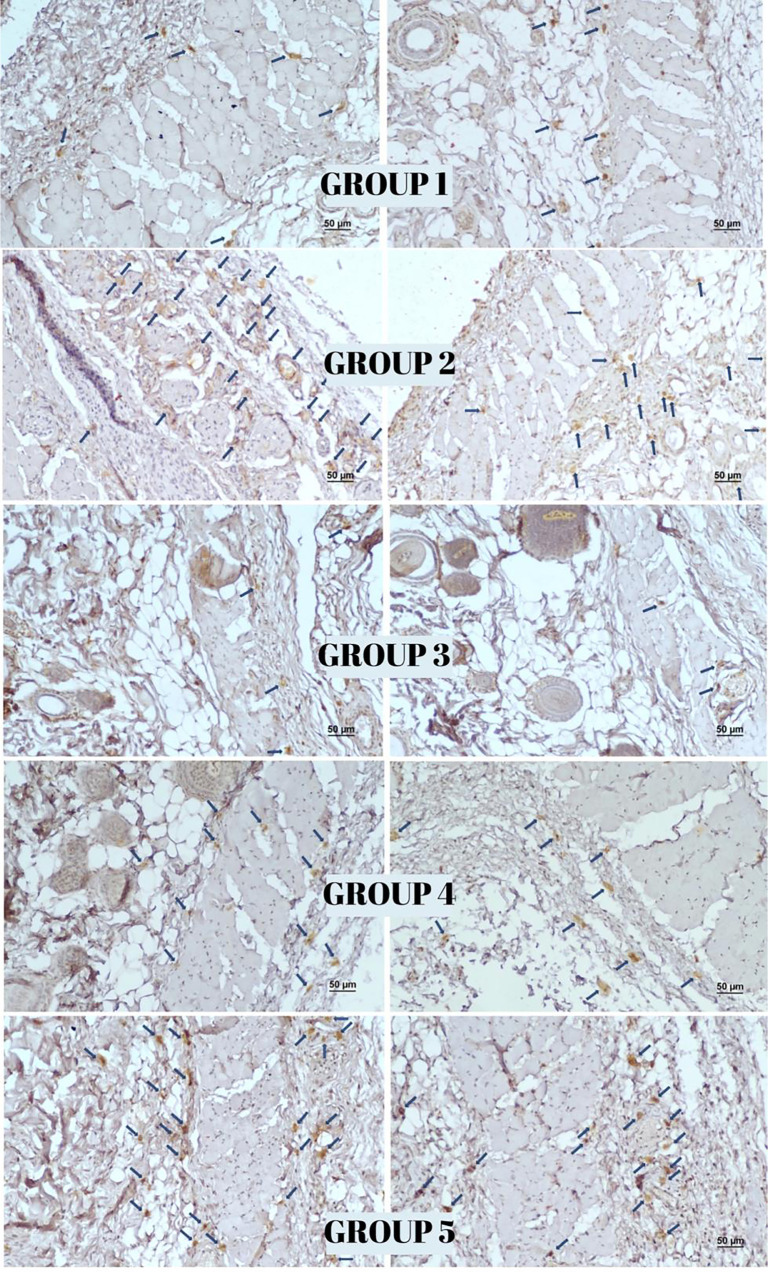
**Immunohistochemical staining for VEGF expression in flap tissues across experimental groups.** Representative micrographs demonstrate differences in staining patterns among groups. (Scale bar: 50 µm; Streptavidin–biotin peroxidase method, 100× magnification). Abbreviation: VEGF: Vascular endothelial growth factor.

**Figure 8.**
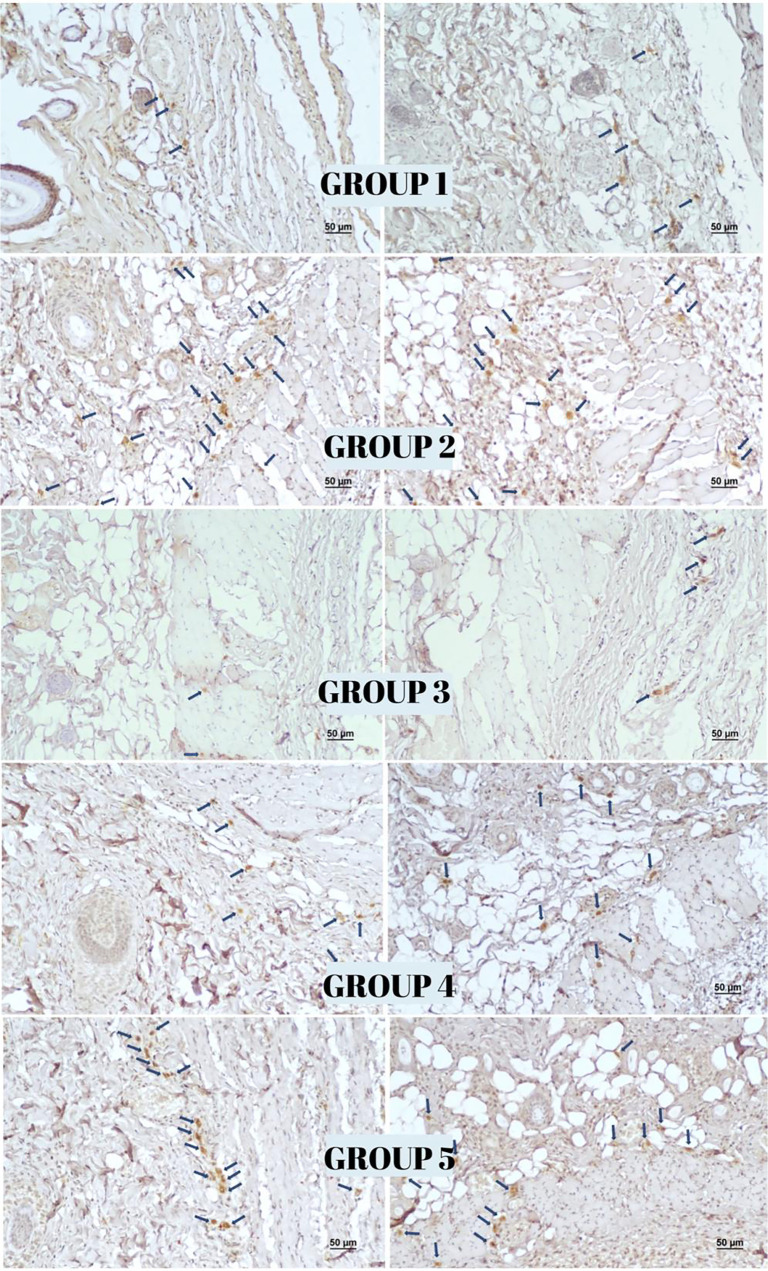
**Immunohistochemical staining for CD34 in flap tissues across experimental groups**. Strong staining intensity is evident in Groups 3 and 5, moderate staining in Group 4, and mild staining in Groups 1 and 2 (Scale bar: 50 µm; Streptavidin–biotin peroxidase method, 100× magnification).

**Figure 9.**
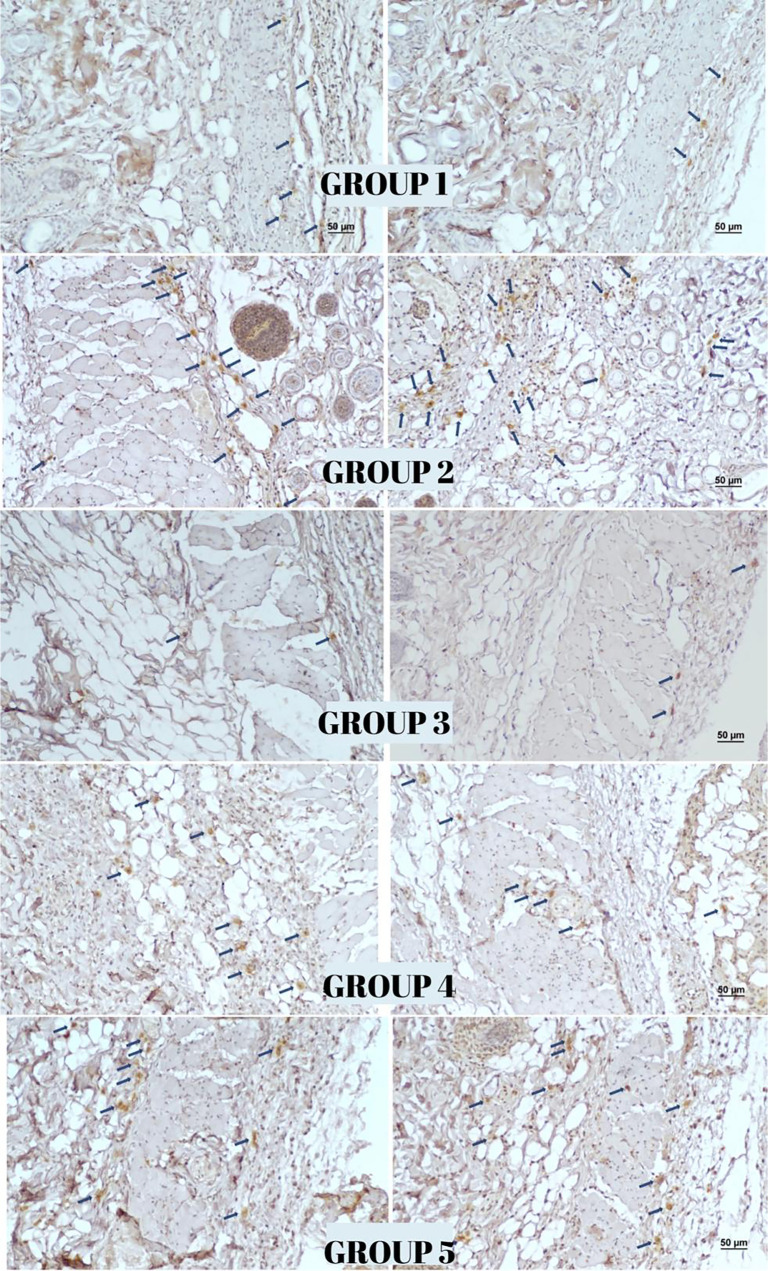
**Immunohistochemical staining for CD105 in flap tissues across experimental groups.** Strong staining intensity is observed in Groups 3 and 5, moderate staining in Group 4, and mild staining in Groups 1 and 2. (Scale bar: 50 µm; Streptavidin–biotin peroxidase method, 100× magnification).

**Figure 10.**
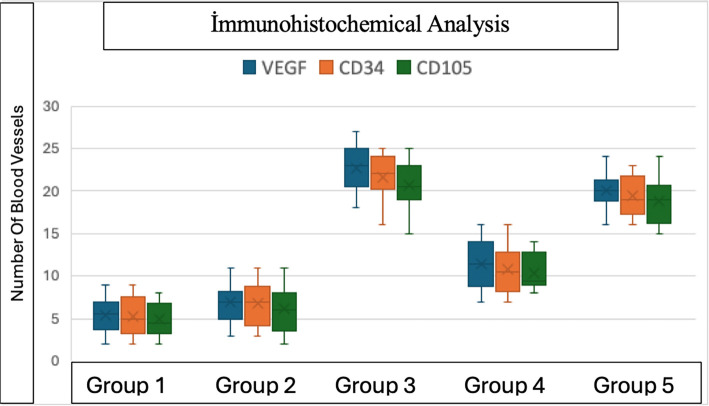
**Graph showing immunohistochemical vascular counts across all groups.** Group 3 demonstrated significantly higher vessel counts compared with Groups 1 and 2, with no significant difference between Groups 1 and 2. Group 5 exhibited significantly higher vascular counts compared with Group 4. Abbreviation: VEGF: Vascular endothelial growth factor.

The highest reactivity for the vessel proliferation markers VEGF, CD34, and CD105 was observed in Group 3, while Group 1 exhibited the lowest reactivity. On day 12, Group 3 demonstrated significantly enhanced angiogenesis compared to both Group 1 (*P* < 0.001) and Group 2 (*P* < 0.001) as assessed through vascularization measurements. A comparison between Group 4 (surgery only) and Group 5 (surgery with topical NTG) revealed a statistically significant increase in vascularization in the NTG -treated group (*P* < 0.001) (see Table 2 and Figures 7–10).

**Table 2:** Mean immunohistochemical vessel counts of the groups, and *P* values of the groups

	**VEGF**	**CD34**	**CD105**
Group 1 (5 day TV+7 day TV)	5,41±2,15 4,04–6,78	5,25±2,17 3,86–6,63	4,91±1,97 3,66–6,17
Group 2 (5 day TN+7 day TV)	6,91±2,42 5,37–8,46	6,75±2,41 5,21–8,28	6,16±2,79 4,39–7,93
Group 3 (5 day TN+7 day TN)	22,66±2,93 20,80–24,53	21,58±2,81 19,79–23,36	20,75±2,66 19,05–22,44
Group 4 (5 day TV)	11,41±3,02 9,49–13,34	10,83±2,79 9,06–12,60	10,41±2,10 9,07–11,75
Group 5 (5 day TN)	20,08±2,35 18,58–21,57	19,41±2,31 17,94–20,88	18,83±2,72 17,10–20,56
	***P* values**		
Group 1-2	1.0	1.0	1.0
Group 1-3	<0.001	<0.001	<0.001
Group 2-3	<0.001	<0.001	<0.001
Group 4-5	<0.001	<0.001	<0.001

Data are presented as mean ± standard deviation and 95% confidence intervals (95% CI). *P* values obtained from one-way ANOVA and < 0.05 was considered statistically significant. Abbreviations: VEGF: Vascular endothelial growth factor; TN: Topical nitroglycerin; TV: Topical vaseline.

**Figure 11.**
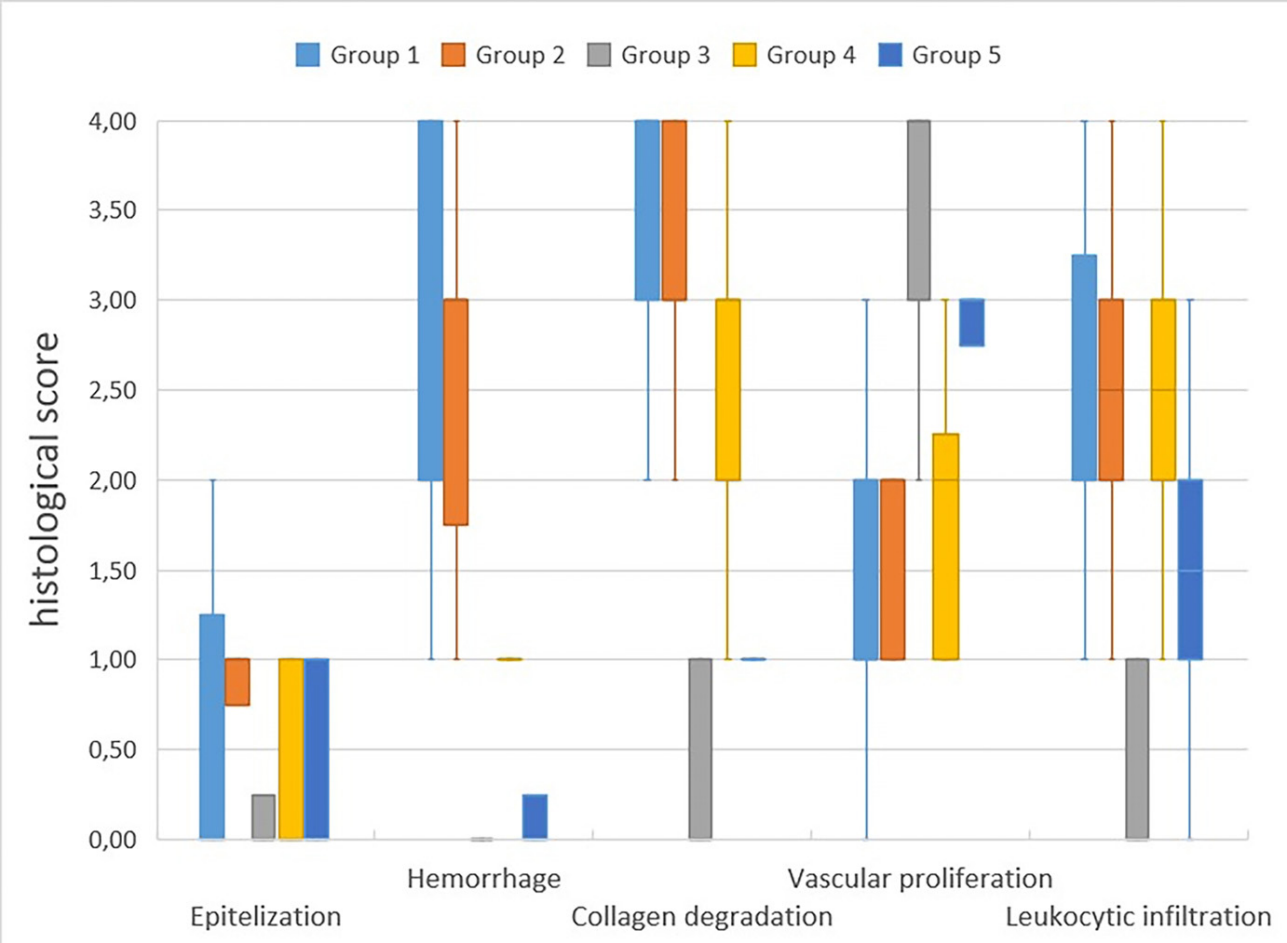
**Graph of histological wound healing scores in all groups.** The evaluated parameters include epithelialization, hemorrhage, collagen degradation, vascular proliferation, and leukocytic infiltration.

Histochemical analyses were conducted using H&E as well as Masson’s Trichrome staining. Five histopathological criteria were evaluated: disruption of epithelialization, hemorrhage, collagen degradation, vascular proliferation, and leukocytic infiltration (see Tables 3 and 4, and Figure 11).

**Table 3:** Histopathological scoring of all groups

	**Disruption in epithelialization**	**Hemorrhage**	**Collagen degradation**	**Vascular proliferation**	**Leukocytic infiltration**
Group 1	1,0(1,5-0)	3(4-2)	4(4-3)	1(2-1)	3(3,5-2)
Group 2	1,0(1-0,5)	3(3-1,5)	3(4-3)	2(2-1)	2,5(3-2)
Group 3	0(0,5-0)	0(0-0)	0(1-0)	4(4-3)	0(1-0)
Group 4	0(1-0)	1(1-1)	1.25(2-1)	2(2,5-1)	2,5(3-2)
Group 5	0(1-0)	0(0,5-0)	1(1-1)	3(3-2,5)	1,5(2-1)
	***P* values**				
Group 1-2	0.999	0.786	0.617	0.842	0.962
Group 1-3	0.171	<.001	<.001	<.001	<.001
Group 2-3	0.102	<.001	<.001	<.001	<.001
Group 4-5	1.000	0.097	0.087	0.097	0.062

Histopathological evaluation of flap tissue in Groups 1–5 using Hematoxylin & Eosin (H&E) and Masson’s Trichrome staining. Five parameters were assessed: Disruption of epithelialization, hemorrhage, collagen degradation, vascular proliferation, and leukocytic infiltration. Data are presented as median and interquartile range (IQR).

**Table 4:** Comparison of histopathological parameters among study groups using the Kruskal–Wallis test with effect size

	***P* value**	**Effect size (ɛ^2^)**	**Interpretation**
Disruption in epithelialization	0.048	0.163	Medium
Hemorrhage	<.001	0.697	Large
Collagen degradation	<.001	0.777	Large
Vascular proliferation	<.001	0.548	Large
Leukocytic infiltration	<.001	0.564	Large

Comparison of five histopathological parameters (disruption of epithelialization, hemorrhage, collagen degradation, vascular proliferation, and leukocytic infiltration) among groups. Effect size is reported as epsilon squared (ɛ^2^), where 0.01 indicates a small effect, 0.08 a medium effect, and 0.26 a large effect. Abbreviation: ɛ^2^: Epsilon squared.

All groups displayed minimal effects on epithelialization. Histopathological evaluation revealed that epithelial disruption was most pronounced in Group 1 (G1: 1.0 [1.5–0.0]) and nearly absent in Group 3, which received prolonged NTG treatment (G3: 0.0 [0.5–0.0]).

Group 1 recorded the highest scores, indicating severe hemorrhage, while Groups 3 and 5 exhibited scores close to normal levels. Both Group 1 and Group 2 demonstrated statistically significant higher levels of hemorrhage compared to Group 3. However, no significant difference was observed between Groups 4 and 5 (*P* ═ 0.097).

The most pronounced tissue degradation was identified in Group 1, followed by Group 2. Conversely, Group 3 demonstrated the lowest degradation scores, indicating histological characteristics most akin to normal tissue. Statistically significant reductions in degradation were observed in Group 3 when compared to Groups 1 and 2. No statistically significant differences were noted between Groups 4 and 5.

The highest level of vascular proliferation occurred in Group 3, whereas Groups 1 and 2 exhibited the lowest levels of proliferation. In Group 3, proliferation was significantly greater than in Groups 1 and 2. Additionally, there were no statistically significant differences between Groups 4 and 5.

Leukocytic infiltration was most prominent in Groups 1, 2, and 4, while Group 3 displayed the least infiltration. Groups 1 and 2 showed statistically significantly higher infiltration compared to Group 3; however, statistical analysis indicated no significant differences between Groups 4 and 5.

## Discussion

In plastic surgery, two-stage skin flaps requiring pedicle division are frequently employed for reconstructing cutaneous defects. Due to factors related to the patient’s overall health and the surgeon’s resources, microvascular procedures may sometimes be impractical. In such instances, shorter and safer pedicled local or distant flaps are preferred. However, patients face various restrictions during the period preceding pedicle division [11], making a reduced interval for pedicle division highly desirable.

In our study, we hypothesized that the application of topical NTG to a pedicled flap harvested from the rat’s back would accelerate the autonomization process—defined as the flap’s ability to survive independently of its pedicle—by promoting angiogenesis along three sides of the flap. Our clinical findings indicated that the group receiving NTG for 5 days prior to pedicle division (Group 2) and the group receiving NTG both before and for an additional 7 days after pedicle division (Group 3) demonstrated significantly higher flap viability compared to the control group treated with vaseline (Group 1). Most studies evaluating flap survival with topical NTG have utilized the McFarlane model, and to date, no study has specifically assessed flap autonomization. Our experimental model is distinctive in its focus on evaluating flap autonomization.

Flap tissue can develop a capillary network with the surrounding recipient tissue, thereby ensuring its survival without dependence on the pedicle—a process known as flap autonomization, which is essential before the pedicle can be safely divided. Clinically, an average interval of approximately 3 weeks is recommended for pedicle division [12]. To enhance flap autonomization and shorten this interval, temporary pedicle compression using silicone or staplers (i.e., physical ischemic preconditioning) is the most commonly employed method [13].

In a 2023 study, Berkane et al. [1] demonstrated using a rat model of superficial inferior epigastric artery skin flaps that the distal, most hypoxic portion of the flap exhibited greater viability following pedicle division, attributing this phenomenon to hypoxia-induced angiogenesis. This finding is consistent with our observation of necrosis occurring in areas adjacent to the pedicle in nearly half of our flaps. The existing literature includes numerous studies exploring the angiogenic effects of various growth factors, such as transforming growth factor (TGF), epidermal growth factor (EGF), fibroblast growth factor (FGF), and VEGF. For instance, Zhang et al. [14] reported that exogenous VEGF enhanced early outcomes of pedicle division and flap survival in a rat tube pedicle flap model; however, due to its potential modulatory role in cancer, the clinical application of exogenous VEGF is currently unfeasible [15].

Another agent investigated for its angiogenic properties is NTG. As a prodrug, NTG is rapidly metabolized in tissues to release NO, a mediator with diverse biological effects. NO activates guanylate cyclase in vascular smooth muscle cells, resulting in increased production of cyclic guanosine monophosphate (cGMP). cGMP subsequently activates protein kinase G, cGMP-dependent ion channels, and cGMP-sensitive phosphodiesterases, with the most notable effect being smooth muscle relaxation through intracellular calcium redistribution [16]. Furthermore, studies have indicated that inhibition of NO synthesis obstructs angiogenesis in models of portal hypertension [17], [18], and that the angiogenic effects of VEGF via its receptor VEGFR-2 are NO-dependent [19]. Consequently, NO-mediated mechanisms have been implicated in VEGF-induced angiogenesis.

Russell et al. [20] utilized sodium nitroprusside as an NO donor in a McFarlane flap model and reported significant improvements in flap survival with both topical and injectable applications. Despite the established role of NO as an angiogenic mediator, there has been no prior investigation into whether NTG can enhance neoangiogenesis and thereby improve flap autonomization. This gap in research provided the rationale for our study, in which we evaluated flap survival by transecting the pedicle of a 3×3 cm dorsal skin flap on day 5.

Historically, topical NTG was first utilized in an animal study by Rohrich et al. in 1984. In this study, a 30 mg dose applied preoperatively and three days postoperatively resulted in improved axial flap viability in both rats and pigs compared to control groups [21]. Commercially, NTG is available in formulations of 0.2%, 0.4%, and 2%. The 0.2% and 0.4% concentrations are prescribed for anal fissures and hemorrhoids, while the 2% formulation, used for angina, is not available for domestic use. Although numerous studies have demonstrated the benefits of NTG, some have failed to show improvements in tissue viability, often employing a single daily dose [22] or low doses (approximately 5 mg) [23]. Given the short duration of NTG ’s effects, we selected a higher concentration readily available in the market. One study even reported no benefit from a single postoperative dose on flaps and grafts [22].

In our experiments, Group 3—receiving topical NTG both before and after pedicle division—exhibited a significantly larger area of viable flap tissue compared to Group 1, which only received NTG for 5 days followed by vaseline. The greater mean surviving flap area observed in Group 3 suggests that continuous application of topical NTG may be more effective in promoting flap viability.

In the immunohistochemical evaluation, despite both Groups 2 and 5 receiving 5 days of topical NTG, Group 5 demonstrated a statistically significant higher vessel count. Two hypotheses may account for this observation: first, while vessel density on day 5 in Group 2 might have been comparable to that in Group 5, the cessation of NTG thereafter could have hindered the complete maturation of nascent angiogenic vessels, leading to a loss of their vascular characteristics. Second, the vasodilatory effect of NTG prior to sacrifice in Group 5 may have made the early angiogenic vessels more distinctly visible upon staining. This is further supported by the observed clinical reduction in flap viability and decreased vessel counts in Group 2.

To isolate the effects of flap elevation and the angiogenic impact of topical NTG, we conducted a comparative analysis of Groups 4 and 5. While immunohistochemical staining revealed superior vessel counts in Group 5, this difference did not reach statistical significance. However, a statistically significant increase in vessel counts was observed in Group 5, indicating that topical NTG promotes neovascularization.

Regarding other histological findings, NTG significantly affected epithelialization across the groups, with a statistically significant difference observed among the repeated measures (*P* ═ 0.048), although the result was close to the threshold of significance. Consistent with the immunohistochemical results, vascular proliferation was most pronounced in Group 3, followed by Group 5. Furthermore, NTG’s well-known anti-inflammatory properties and its role in preventing ischemia-reperfusion injury were evidenced by a statistically significant reduction in inflammatory cell infiltration in Groups 3 and 5 compared to the other groups [24]. Collagen degradation—a marker of ischemia-reperfusion injury—was significantly higher in Groups 1 and 2, where NTG was not administered post-pedicle division.

While immunohistochemistry provided valuable insights into angiogenesis, the incorporation of molecular techniques—such as *VEGF* gene expression profiling via RT-PCR or the assessment of other angiogenic markers—could enhance the robustness of the findings. These methods are recommended for future research.

## Conclusion

In summary, our findings indicate that topical NTG enhances flap autonomization by promoting angiogenesis, thereby improving flap viability and potentially reducing the time required before safe pedicle division in reconstructive procedures.
